# Atypical event-related potentials revealed during the passive parts of a Go-NoGo task in autism spectrum disorder: a case-control study

**DOI:** 10.1186/s13229-019-0259-3

**Published:** 2019-03-05

**Authors:** Anne L. Høyland, Terje Nærland, Morten Engstrøm, Tonje Torske, Stian Lydersen, Ole A. Andreassen

**Affiliations:** 1 Department of Mental Health, Faculty of Medicine and Health Sciences, Regional Centre for Child and Youth Mental Health and Child Welfare, Norwegian University of Science and Technology, Klostergata 46, N-7030 Trondheim, Norway; 2 Department of Pediatrics, St. Olavs hospital, Trondheim University Hospital, Trondheim, Norway; 3 NevSom, Department of Rare Disorders and Disabilities, Oslo University Hospital, Oslo, Norway; 4 NORMENT, KG Jebsen Centre for Psychosis Research, University of Oslo, Oslo, Norway; 5 Department of Neurology and Clinical Neurophysiology, St. Olavs Hospital, Trondheim University Hospital, Trondheim, Norway; 6 Department of Neuromedicine and Movement Science, Norwegian University of Science and Technology, Trondheim, Norway; 7 Division of Mental Health and Addiction, Vestre Viken Hospital Trust, Drammen, Norway; 8 Division of Mental Health and Addiction, Oslo University Hospital, Oslo, Norway

**Keywords:** ASD, ERP, Passive condition, N1, P3a

## Abstract

**Background:**

The core features of autism spectrum disorder (ASD) are easily recognizable in non-structured clinical and real-life situations. The features are often difficult to capture in structured laboratory settings, and the results from tests do not necessarily reflect symptom severity. We investigated neurophysiological processing in the *passive* parts of a cued Go-NoGo task, using the *active* parts of the test as a comparator.

**Methods:**

Forty-nine adolescents diagnosed with ASD and 49 typically developing (TD) adolescents (age 12–21 years) were included. Daily life executive function was assessed with the Behavior Rating Inventory of Executive Function (BRIEF). We applied a visual cued Go-NoGo task and recorded event-related potentials (ERPs). We investigated occipital N1, a component related to early perception of visual stimuli, and P3a, a fronto-central component related to switching of attention, in the passive and active parts of the test.

**Results:**

During the passive parts, the ASD group had statistically significantly longer N1 latency (*p* < 0.001, Cohens *d =* 0.75) and enhanced amplitude of P3a (*p* = 0.002, Cohens *d =* 0.64) compared to the TD, while no significant differences were observed in the active parts. Both components correlated significantly with the Behavioral Regulation Index of the BRIEF (partial correlation *r* = 0.35, *p* = 0.003).

**Conclusion:**

Delayed N1 response, indicating altered visual perception, and enhanced P3a response, indicating increased neural activation related to attention allocation, were found during the passive parts of a Go-NoGo task in ASD participants. These abnormal ERP signals in the non-structured settings were associated with everyday executive function, suggesting that neurophysiolocal measures related to atypical control of alertness and “hyper-awareness” underlie daily life dysfunction in ASD. Assessments during passive settings have a potential to reveal core neurobiological substrates of ASD.

**Electronic supplementary material:**

The online version of this article (10.1186/s13229-019-0259-3) contains supplementary material, which is available to authorized users.

## Background

Autism spectrum disorder (ASD) is a neurodevelopmental disorder with impaired reciprocal interaction and a restricted pattern of behavior [1, 2]. ASD is now widely accepted as a developmental neurobiological disorder with multifaceted etiology comprising genetic, environmental, and gene-by-environment influences resulting in perturbations in early brain development [3, 4]. Despite clear central nervous system involvement, ASD is still purely behaviorally defined. The core behavioral features of ASD are highly context-dependent, and assessment of the behaviors that define ASD has proven difficult to do in a stringent and controlled manner. Typical behavior is usually more apparent in complex real-life situations than in standardized laboratory settings [5], suggesting that un-structured or passive test situations are needed to reveal neurophysiological substrate for ASD.

Constant adaption to potentially relevant environmental events balanced against attention modulated by expectation and desire or relevance is necessary to achieve goal-directed behaviors [6, 7]. The ability to control sensory responsiveness through gating mechanisms, i.e., filtering irrelevant or interfering stimuli or impulses, is fundamental for normal functioning [8]. Such information processing mediating selectivity is defined as attention [9–13]. Atypical attention modulation is suggested as a basic factor in the development of core features in ASD [9, 14]. Atypical sensory processing is repeatedly described [15, 16] and may reflect core neuropathology underlying clinical symptoms of ASD. Sensory processing dysfunction is common [17–20] and found in as much as 90% of children with ASD [21]. Obligatory processing of task-irrelevant stimuli seems to be a hallmark of autistic cognitive style, probably as a result of insufficient top-down filtering [12, 22]. Several studies support that individuals with ASD manifest unusual neural responses to task-irrelevant features [6, 22–25], suggesting that passive or unstructured test settings can reveal relevant neurobiological alterations.

Studies on attention in ASD compared to typical developing (TD) yield mixed results both regarding performance and neurophysiological responses [26–29]. Both reduced and enhanced neural activation is found in novelty-processing [25, 30–32]. This is suggested dependent of experiment context and seems different for active (requiring a response) or passive (no response) novelty detection. Keehn et al. found increased neural activation in ASD when given an active task but reduced processing during passive tasks [9]. Deviant processing during passive tasks could be related to atypical control of alertness, over-focusing, and maladaptive attention allocation.

Event-related potentials (ERPs) are small voltage oscillations measured at the scalp that are time-locked to the processing of external events and can give insight to neurobiological processes involved in cognition [33]. There are numerous ERP studies in ASD, but there is a large degree of variation in the findings. The occipital N1 wave, an early response to visual stimuli, is modulated by selective attention [34]. The N1 amplitude is enhanced in discrimination tasks [35], and studies have found alterations in the visual evoked potentials in ASD [36]. The P3 has an early, fronto-central component, P3a, and a later and more posterior component P3b [37, 38] and is elicited whenever a task conveyed contextual information about an impending change [39, 40]. In a meta-analysis of the P3 components, Cui et al. [41] reported a tendency of reduced P3b amplitude among ASD subjects. A striking feature in their analysis is the heterogeneity among the 32 studies included. Differences in amplitudes and latencies are attributed to variances in paradigm and the heterogeneity among ASD participants. Another component related to change detection is the mismatch negativity (MMN), typically elicited by auditory stimuli. Analogous response occurs also in other sensory modalities [42]. Findings from studies of MMN in ASD have been inconsistent. As in studies on P3, both amplitudes and latencies are found to be significantly larger [43], in the normal range [44] and reduced [31, 45]. However, few studies have focused on ERPs during unstructured sessions of the cognitive experimental procedures.

Difficulties with various aspects of executive function (EF) in everyday life among ASD subjects are consistently observed by clinicians and family members [46–48]. The disparity between neuropsychological testing of EF and real-life EF is well known [5, 46, 49, 50]. Neuropsychological examination is usually conducted in a controlled, quiet, and structured setting, while the real world is complex, noisy, unstructured, and at times even chaotic [50]. Thus, a questionnaire for EF assessment in real-life setting, the Behavior Rating Inventory of EF (BRIEF), was developed [51]. BRIEF is suggested to capture distinct patterns of EF problems in individuals with ASD [52, 53]. The cued Go-NoGo task is an experimental task that enables the study of different executive control processes as attention, reaction time, and inhibition of pre-potent responses [54]. The *active* part of the test is when the participant either has to respond *or* inhibit a response to a stimulus. The *passive* parts are present after stimuli, when the participant *neither* needs to respond *nor* inhibit a response. Although the passive parts may be differently related to the cognitive task, we propose that it could reveal important electrophysiological abnormalities of ASD since core autistic characteristics are particularly revealed during unstructured settings.

We have previously reported no difference in behavior performance among ASD and controls in a Go-NoGo task [28] and similar ERPs during the active parts of the test [29]. However, the parents rating on the BRIEF indicate significant EF difficulties in the ASD group. Thus, our test results in a structured experiment of executive control were not consistent with the reported real-life EF problems [50]. However, the passive parts of the Go-NoGo task may represent a setting that better mimicks the unstructured real-life situations where abnormalities are well documented. This is supported by several studies reporting unusual neural responses to task-irrelevant features in ASD [6, 22–25]. The novel approach of the current study was to investigate the ERPs from both the non-active “breaks” (passive parts*)* of the Go-NoGo task and in the same test from the standard “task” parts (active). Few studies have investigated this aspect of ERPs, as most test paradigms used in ASD are based on cognitive challenges and processing effort. We hypothesize that important information about the core neurobiological substrate of ASD could be revealed in the passive parts of the Go-NoGo task. The aim of the present study was to identify differences in the electrophysiological processing in adolescents with ASD compared to typical developing adolescents (TD) during the passive parts of the Go-NoGo task and investigate if these ERP components were associated with real-life EF measured with the BRIEF indexes. We hypothesized that ASD group would show abnormal ERPs related to early visual processing (N1) and attention allocation (P3a) during the passive parts of the Go-NoGo task, since this represents an unstructured setting that better reflects the real-life situation.

## Materials and methods

### Participants

Fifty adolescents with a confirmed diagnosis of ASD without intellectual disability from outpatients attending St. Olavs Hospital, Trondheim, Norway, were included in the study during 2013–2016. The sample consisted of 13 girls and 37 boys, aged 12–21 years, average 15.6 years.

Forty-nine typically developing adolescents, matched for age and gender, were recruited from adjacent schools through invitations/bulletins to all students/parents. In the invitation letter and recruitment posts, we invited healthy adolescents. The parents confirmed in writing that their child did not suffer from any chronic disease or psychiatric problems presently or previously. Eighteen girls and 31 boys from 12 to 20 years were included, average 15.6 years.

The ASD patients were diagnosed according to the ICD-10 [1] F.84 criteria for pervasive developmental disorder based on developmental information and clinical assessments. To identify characteristics associated with ASD, the parents of all participants completed the lifetime version of the Social Communication Questionnaire (SCQ) [55]. This questionnaire is based on the Autism Diagnostic Interview-Revised (ADI-R) [56] and is found valid for the ASD diagnosis [57, 58]. ADI-R has been found to discriminate well between ASD and non-ASD [59]. The ASD group in our study had markedly increased scores on SCQ compared with TD (*p* < 0.001).

Eighteen (37%) individuals in the ASD group had neuropsychiatric comorbidity, all but one with attention problems (Attention Deficit Disorder with or without hyperactivity (ADHD)). Eight (16%) had more than one comorbid diagnosis. Six (12%) had a diagnosis of epilepsy, all but one with co-occurring ADHD. Twelve (25%) of the ASD individuals used medication regularly, four were on stimulants, two used atomoxetine and the six participants with epilepsy were on antiepileptic medication.

Intelligence quotients (IQs) were registered in the ASD group. The IQs were mainly obtained during clinical assessments prior to the current study. Most of the participants were tested using the Wechsler tests [60], while one participant was assessed using the Leiter test [61] because of specific language problems. Some participants were tested after recruitment into the current study, applying the Wechsler Abbreviated Scales of Intelligence [62]. When the difference between verbal and performance IQs was ≥ 30, we did not calculate full-scale IQ (FIQ). To be included in the study, verbal (VIQ) or performance IQ (PIQ) had to be within the normal variation (≥ 70).

EF in everyday life were measured using the BRIEF [51]. The BRIEF contains eight clinical scales that are grouped in a Behavioral Regulation Index (BRI) and a Metacognition Index (MI). The BRI comprises the child’s ability to modulate both behavior and emotional control and the ability to move flexible from one activity to another. The MI is associated with the ability for active problem solving and to initiate, organize, and monitor their own actions [51]. The Global Executive Composite (GEC) is a summary score that incorporates all eight clinical scales. T-scores of ≥ 65 are considered to represent clinically significant areas. The ASD group showed significantly increased GEC (mean (SD), ASD 67.8 (10.2), TD 42.2 (6.4), *p* < 0.001, Cohens *d* = 2.9).

One of the participants in the ASD group had more than 70% omissions/commissions in the performance data of the test and was excluded. The others, 49 ASD individuals and 49 TD, were included in the study.

For detailed demographic information of the sample, see Additional file 1: Table S1.

### Experimental task, electrophysiological recording, and analysis

#### Experimental task

We used a visual-cued Go-NoGo task [63]. The categories of visual stimuli (see Additional file 1: Figure S1, http://www.mitsar-medical.com/eeg-software/qeeg-software/download.html) included 15 pictures of each category: animals, plants, and humans. All participants completed 300 trials. Each trial consisted of a subsequent pair of stimuli (S1 and S2). When S1 was a cue, the subsequent S2 might require a response and the recording after the S2 was assigned the active part of the test. The passive parts of the test were all situations not requiring a response, that means after all S1 and after S2 when S1 was a non-cue (plant). In the non-cue trials, 50% of the S2 were accompanied by a novel sound. The combination of auditory and visual stimuli complicated our interpretation of ERPs and we excluded the results from this part of the test. S1 and S2 were presented for 100 ms with an 1100 ms inter-stimulus interval and an inter-trial interval of 3000 ms. The trials were grouped into blocks separated by short breaks. In each block, a unique set of five pictures from each picture category was selected. Each block consisted of a pseudo-random presentation of 100 stimulus pairs with equal probability for each trial category. The participants were instructed to respond by pressing a button with their right index finger as quickly as possible without making mistakes in all Go trials and otherwise refrain from responding. They were also instructed that a sound would turn up that they should ignore.

During the task, participants were seated in a comfortable chair 1.2 m from the computer screen. The pictures (size approximately 20 × 15 cm) were presented in the middle of an 18-in monitor using the Psytask (http://www.mitsar-medical.com/eeg-software/qeeg-software/download.html)) software (from Bio-medical, Clinton Township, Michigan USA). The time interval from the presentation of the second stimulus to the response, reaction time (RT), and intra-individual reaction time variability (RTV) was registered by the software. The ERPs were averaged through trials with correct responses. The software also registered omissions and commissions. Task performance and ERPs are previously reported (Table 1), for more details, see Høyland et al. [28].

**Table 1:** Performance in task and ERPs in a visual-cued Go-NoGo task [29]

	TD	ASD		
	Mean (SD)	Mean (SD)	*p* value	Cohen’s *d*
*n*	49	49		
Reaction time	330.5 (62.0)	338.7 (65.2)	0.53	0.13
RTV	10.0 (3.7)	9.9 (3.6)	0.86	0.04
Omissions	3.5 (3.9)	3.7 (4.9)	0.75	0.06
Comissions	1.5 (1.9)	1.5 (1.9)	0.96	0
Cue P3 (Pz)	4.41 (2.26)	4.41 (2.83)	0.99	0
P3 NoGo (Cz)	11.66 (4.18)	11.94 (5.99)	0.92	0.05
P3 Go (Pz)	9.24 (4.15)	9.36 (3.00)	0.36	0.03

#### Electrophysiological recordings

Electroencephalogram (EEG) was recorded using a Mitsar (http://www.mitsar-medical.com) EEG system with a 19-channel tin electrode cap (Electro-cap International, Eaton, OH, USA). The electrodes were placed according to the international 10-20-system. The input signals were referenced to earlobe electrodes and filtered between 0.5 Hz and 50 Hz and digitized at a sampling rate of 500 Hz. Impedance was kept below 5 kΩ for all electrodes. Quantitative data were obtained from the WinEEG software (http://www.mitsar-medical.com) in common average montage prior to data processing. Eye blink artifacts were corrected by zeroing the activation curves of individual independent components corresponding to eye blinks. In addition, epochs of the filtered EEG with excessive amplitude (> 100 μV) and/or slow (> 50 μV in the 0–1 Hz-band) and excessive fast (> 35 μV in the 20–35 Hz-band) frequency activity were automatically excluded from further analysis. All participants also had a six-minute resting EEG registration and a specialist in clinical neurophysiology examined the recordings and found no epileptic activity.

**Fig. 1:**
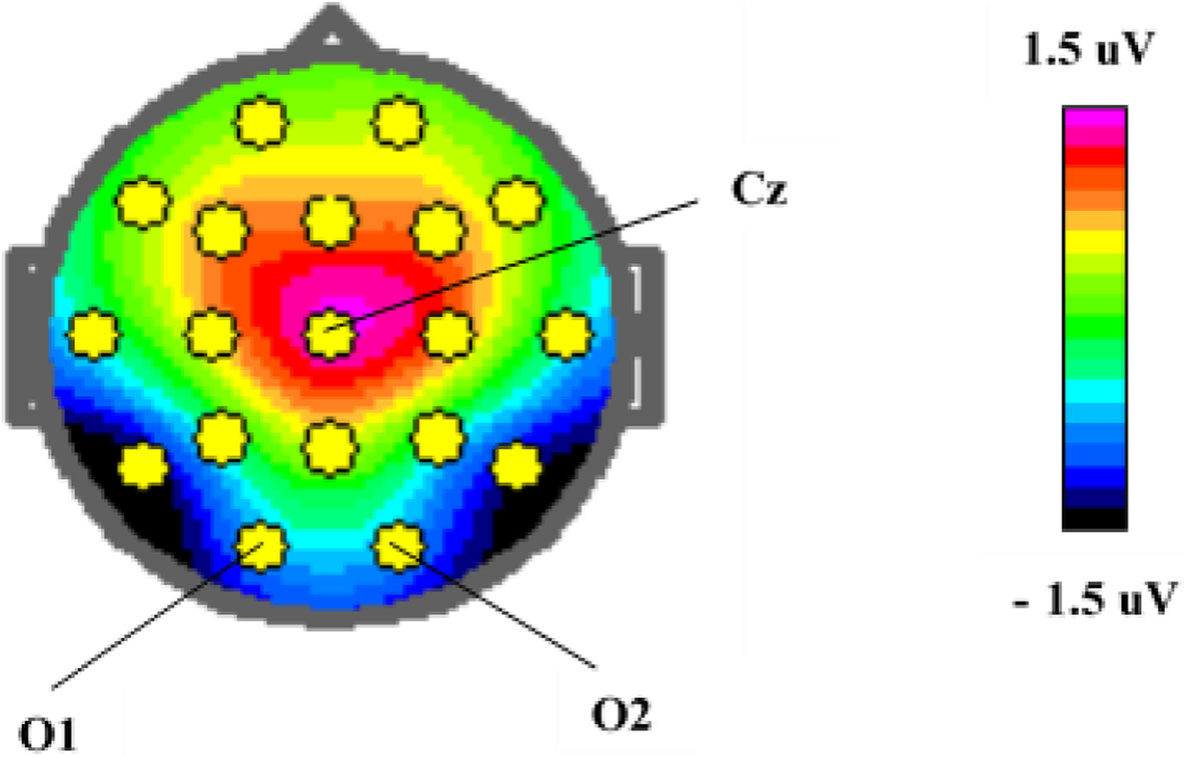
Brain mapping of P3a amplitude visualized as difference between ASD and TD, 174 ms after non-cue stimulus 1

The ERPs for each individual were based on averaging the trials of the respective task condition with correct response after artifact correction. The number of artifact-free trials averaged were (mean (± SD, range)) TD 269 (± 22.4, 191–300), ASD 261 (± 37.9, 109–295). This makes a non-significant difference in averaged trials. The ERPs were measured by convention; N1 as the averaged peak amplitude and latency through O1 and O2 and P3a as mean amplitude in Cz, both in time window chosen from the grand average file for all participants (Table 2). The topography of the P3a component is illustrated in Fig. 1. The paradigm in our study does not involve a sequence of repetitive stimuli and does not elicit a MMN-component.

**Table 2:** Event-related potentials (ERPs)—recordings

	Time window	Electrodes
N1 peak amplitude and latency	140–210 ms	O1 and O2
P3a mean amplitude	250–320 ms	Cz

*Cz* central midline electrode

*O1*/*2* respectively left and right occipital electrode

### Study design and outcomes

The primary outcome for the current study was the ERPs N1 and P3a elicited during passive conditions in a visual cued Go-NoGo task. We also investigated if these ERPs were associated to executive function as measured by the BRIEF.

### Statistical analysis

We compared the N1 amplitude, N1 latency, and mean P3a amplitude between ASD and TD using Student’s *t* test. In addition, we adjusted these analyses for age using linear regression, giving practically identical results (data not shown). We calculated a receiver operating characteristic (ROC) curve to estimate the sensitivity and specificity of the analyzed ERPs. We calculated the correlation between the ERP components and age using the Pearsons correlation coefficient. We also calculated the partial correlation between these ERP components and the BRIEF indexes adjusted for age.

Normality of residuals was checked by visual inspection of Q-Q plots. Statistical analyses were carried out in IBM SPSS Statistics 25.0. Since these analyses are based on incidental discovery in the first analysis of the current experiment and sample, we adjusted the *p* values using the Benjamini-Hochberg procedure [64] to preserve the false discovery rate (FDR).

## Results

**Fig. 2:**
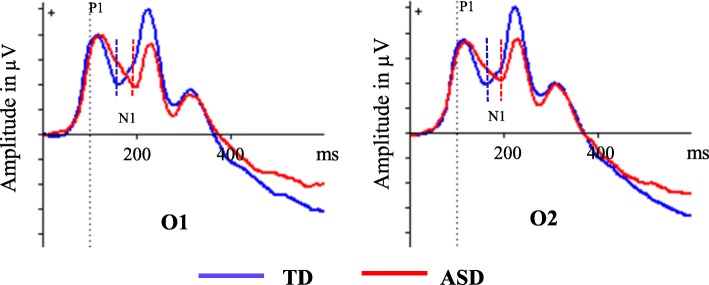
Event-related potentials in left (O1) and right (O2) occipital electrodes after non-cue stimulus 1 in autism spectrum disorders (ASD) and typically developing (TD). Amplitude in microvolts (μV), latency in milliseconds (ms)

**Fig. 3:**
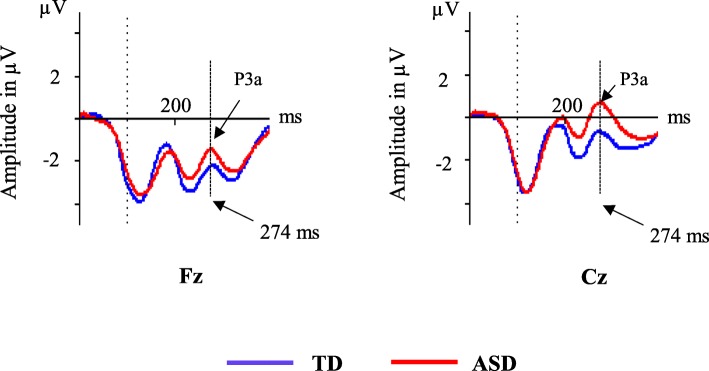
Event-related potentials in central midline electrodes (Fz and Cz) after non-cue Stimulus 1 in autism spectrum disorders (ASD) and typically developing (TD). Amplitude in microvolts (μV), time in milliseconds (ms)

During the passive conditions, the N1 latency and the mean P3a amplitude were significantly increased in ASD (Table 3, Figs. 2 and 3). This was independent of stimulus relevance and in all situations where no action was required (passive parts), i.e., after both cue and non-cue S1 and passive S2, pointing in the direction of a more basic perceptual disturbance. By visual inspection of the grand average file, the P1 component seemed similar in the two groups (see Fig. 2). We also investigated the potential P3b after non-cue S1 and found no significant difference (see Additional file 1: Table S4). The N1 was dependent of age, with negative correlations in both TD and ASD (TD *r* = − 0.41, *p* = 0.004; ASD *r =* 0.52, *p* < 0.001), P3a showed no correlation with age (TD *r* = 0.06, *p* = 0.66; ASD *r =* 0.03, *p* = 0.86). The N1 amplitudes showed no significant differences between the ASD and TD groups in any of the recorded conditions (Table 3). The P3a has, as shown from the mapping (see Fig. 1), a distribution fronto-centrally, with main peak around Cz.

**Table 3:** N1 and P3a in *passive* parts of the test

		TD	ASD		
		Mean (SD)	Mean (SD)		
		*n* = 49	*n* = 49	*p* value	Cohen’s *d*
Passive phase after non-cue S1 (S1 = plant)	N1 amplitude^1^	2.30 (4.86)	1.77 (5.13)	0.66	0.11
	N1 latency^1^	168.3 (15.7)	181.3 (18.9)	0.003*	0.75
	Mean P3a Cz	− 0.99 (1.91)	0.25 (1.99)	0.005*	0.64
	Mean P3a Fz	− 2.57 (1.71)	− 1.93 (1.92)	0.14	0.35
Passive phase after S2 (S1 *and* S2 = plant)	N1 amplitude^1^	2.37 (5.42)	1.72 (5.10)	0.66	0.12
	N1 latency^1^	165.4 (16.5)	179.3 (19.5)	0.003*	0.77
	Mean P3a Cz	− 0.86 (1.87)	0.35 (2.46)	0.014*	0.55
	Mean P3a Fz	− 3.00 (2.1)	− 1.54 (2.0)	0.003*	0.82
Passive phase after cue S1 (S1 = animal)	N1 amplitude	2.39 (4.95)	1.95 (4.91)	0.66	0.09
	N1 latency	163.9 (15.4)	179.6 (20.5)	0.003*	0.87
	Mean P3a Cz	− 1.44 (1.85)	− 0.36 (2.34)	0.024*	0.51
	Mean P3a Fz	− 1.29 (1.93)	− 0.83 (1.85)	0.31	0.24

^1^Amplitude in microvolts and latency in milliseconds

*Benjamini-Hochberg adjusted *p* values, significant at 0.05 level after adjustment for 12 hypotheses

The data of N1 from O1 and O2 are averaged

**Fig. 4:**
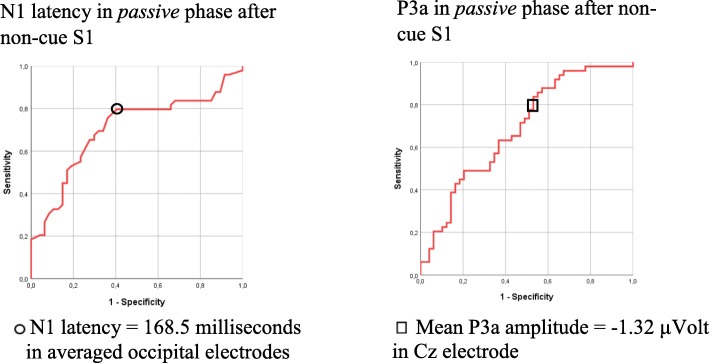
ROC curves for N1 latency and P3a amplitude in *passive* phase after non-cue S1

The ROC curve showed an area under curve (AUC) of 0.7 for both components, see Fig. 4. We chose cut points giving sensitivities as close to 80% as possible. We obtain a sensitivity of 80% and a specificity of 60% when setting the cut-off point of N1 to 168.5 ms. Correspondingly, setting the cut-off point of P3a to − 1.32 μV gives a sensitivity of 80% and a specificity of 47%.

Both N1 latency and P3a were positively correlated with parent-rated measures of everyday executive function (BRIEF) adjusted for age. When splitting into the indexes BRI and MI, we found a highly significant correlation between these ERP components and the BRI index, see Table 4.

**Table 4:** Partial correlations of ERPs in passive phase after non-cue S1 with BRIEF included indexes, controlled for age

	BRIEF total	BRI	MI
*n*	73	74	74
N1 latency	0.29	0.35*	0.24
P3a	0.21	0.35*	0.11

*Significant at 0.01-level

BRIEF total score and indexes. Behavior Regulation Index (BRI) and Metacognition Index (MI)

The relation between N1 latency/P3a amplitude and BRI including ASD subtypes are illustrated in Additional file 1: Figures S2 and S3. The ASD group included participants with comorbid ADHD. The case-control differences in ERPs and the correlations to BRIEF were substantially the same after excluding these participants, see Additional file 1: Tables S2 and S3.

We also investigated the same ERP components in a corresponding test with pictures of emotional faces instead of animals/plants and obtained substantially the same findings, see Additional file 1: Table S4.

## Discussion

The main finding of the present study was significant case-control ERP differences in a cued Go-NoGo task during the passive parts of the test. The ASD group had a delayed occipital N1-component and increased fronto-central P3a-amplitude in the passive parts of the task, while results in the active part were without significant differences in line with previous reports [28, 29]. These abnormal ERP signals were associated with everyday executive function, suggesting that neurophysiolocal measures related to atypical control of alertness and “hyper-awareness” underlie daily life dysfunction in ASD. Taken together, the present findings suggest that assessments during passive experimental settings reveal core neurobiological substrates of ASD.

Executive dysfunction in everyday life is typical for ASD subjects and contributes substantially to the degree of disability [46–48]. However, in the laboratory, both performance and electrophysiological measures of EF may be equal to TD adolescents [5, 27, 29]. Interestingly, in the passive part of the cued Go-NoGo task, the ASD group differed from the controls. The finding that significant case control differences are revealed when no action is triggered (passive parts) is consistent with parental and clinicians’ experience of the increased struggles many individuals with ASD show in non-structured settings, which is a core feature of social human interactions. Thus, the current findings suggest that the identified atypical neurophysiological mechanisms may also underlie everyday dysfunction in ASD. Lastly, it is possible that the findings during the passive part of the neuropsychological test may be a promising candidate for biomarker of ASD which may have a potential as assessment of change.

Our findings are in line with the assertion that abnormal processing stimuli is a key feature of ASD cognitive style [22]. This suggests that ASD individuals have particular problems with the ability to gate sensory information. Keehn et al. [9] discuss how aberrant attentional mechanisms can be linked to the emergence of core ASD symptoms. Top-down modulation of attention in active or passive tasks influence levels of alertness and, thus, processing of new information [9]. The increased N1-latency might reflect increased attentional load and processing effort of both important and unimportant stimuli, and be a neurophysiological correlate of “hyper-awareness,” impairing the ability to suppress irrelevant or interfering stimuli as described by Garavan [8]. In real life, this can reflect difficulties in ignoring distracting information. Friedman and Miyake [65] sought to examine the association between inhibition related functions and found that resistance to distractor interference was closely related with other components of everyday EF, such as task-switching ability and cognitive failures. An increased P3a amplitude mirrors more effort involved in allocation or orientation to novelty and change. Restrictive and repetitive behavior is one of the two diagnostic domains in ASD-criteria in DSM-5 [2] and one of the most striking clinical features of the neurodevelopmental disorder. The resistance to change and insistence of sameness, aspects of repetitive behavior, may very well be coping mechanisms to aberrant perception.

Visual stimuli evoke neural activity in the visual cortex that would be captured by occipital electrodes. Classification of the stimulus is obligatory to obtain acceptable performance in the Go-NoGo task, initiating top-down attentional influence on this early visual component. The N1-attention effect is shown to be the same for both target and non-target stimuli, consistent with a simple modulation of feedforward sensory activity [66]. Attention to stimuli, and not passive watching, enhances N1 amplitude [66] and is expected to affect N1 in our study. We did not find differences in N1-amplitude, reflecting similar response in both groups. Reduced sensory gating might contribute to sensory overload and experienced hypersensitivity [67]. Increased N1-latency may be associated with greater complexity [68, 69], demonstrating an association between latency and processing effort. Enhanced N1-latency may therefore reveal both aberrant sensory processing and altered attentional effect. We found age-related changes of N1-latency in the same range in both TD and ASD, indicating similar maturation processes.

The fronto-central P3a-component is described to reflect orientation to information about an impending change in the task [39]. Previous findings on P3a in ASD are inconsistent. Gomot et al. [32] described enhanced P3a in the ASD group, while Jeste and Nelson found reduced P3a [70]. Our task requires discrimination, and thus, a P3a is expected in all participants. In contrast to Keehn et al. [9], P3a showed a correspondingly significantly enhanced amplitude in all passive conditions in ASD in our study. The P3b elicited in the active parts (Go and NoGo) were similar between the two groups of participants [29]. Also the MMN literature has divergent findings in the ASD group, both enhanced and normal and reduced MMN is described [45]. Both P3a and MMN are linked to sensory processing [32]. The coexistence of atypical sensory processing and deviant attentional salience detection and responses to change will cultivate need of predictability and, thus, insistence of sameness and resistance to change [30, 31].

Our findings are divergent from the recent meta-analysis of P300 in ASD, where Cui et al. [41] report no differences in P3a amplitude. Variances in paradigm and also whether recording in the active or passive condition may contribute to this divergence [9]. In Adam and Jarrolds study of inhibition [24], they found that children with ASD had difficulties inhibiting irrelevant stimuli but not pre-potent responses, indicating a greater tendency to process interfering distractors.

As described in the “Materials and methods” section, our ASD sample included 18 individuals with neuropsychiatric comorbidity. This is in line with other studies of comorbidity in ASD [71]. A range of psychiatric conditions are found to show altered ERPs [72, 73], and disentangling the effects of comorbidity is necessary. In our previous study [29], we reported increased response preparation and enhanced conflict monitoring in adolescents with ASD during a Go-NoGo task. We interpreted this as a possible link to the feature “Insistence of sameness”, but it may also be related to enhanced performance and superior academic skills [74]. This was also found only in adolescents older than 16 years or participants without comorbid ADHD and may, therefore, be linked to subgroups of ASD. Inconsistent with the results in our previous paper [28, 29], excluding the participants with ADHD or specific age groups did not affect N1 latency and P3a amplitude. This may indicate that the abnormalities related to sensory perception and novelty detection are more closely connected to core features of ASD and less to concurrent developmental disorders.

The sensitivity and specificity of classification results based on the components N1 and P3 suggests that the current findings should be replicated in independent samples. Thus, if the paradigm is further improved, these ERPs may be developed into biomarkers of ASD. There is a clear need for direct and stringent assessments of ASD that can be used as a measure of change; the ERPs elicited in this procedure may be a promising candidate for such assessment.

We found a positive correlation of ERPs related to perception and attention orientation and BRIEF, most markedly to the BRI. BRI comprises the ability to modulate both behavior and emotional control which very well can be affected by the hyper-awareness related to aberrant perception and attention allocation. The correlations between ERP components and BRIEF scores were weak (*r* = 0.35). As suggested by the Additional file 1: Figures S2 and S3, this is probably due to a high variance among different subgroups. The presence of subgroups may induce noise and confound the present results. However, it may also indicate interesting phenomena related to the pathophysiology of ASD which should be investigated in larger studies with statistical power to reliably identify relevant subgroups. Other aspects of EF as inhibition of prepotent responses in ADHD will also contribute to BRI and affect the variability of the results. Cognitive aspects captured by the MI seem more spared in ASD and are less correlated to these ERPs.

## Strengths and limitations of the study

All participants were tested by the same technician in the same lab to reduce variations caused by testing conditions. All participants initially included except one completed the task to satisfaction and was kept in the final sample. The results presented were based on an unexpected finding in a secondary analyses and the complexity of the task used is not optimal for this research question. However, the findings are strong and there is no reason to doubt the main outcome of the study. The results should be replicated in independent samples using a simpler and more targeted paradigm. As the studies in this research-field have provided large differences in mean and SD, with a large variation in methodology, we were not able to perform any power-analysis. Prior to the study, we decided to include 50 ASD adolescents and 50 matched controls based on reported studies applying similar ERP experimental paradigms and methodology [54]. We included patients previously diagnosed with ASD, but did not repeat the diagnostic assessment. However, we underpinned the diagnosis by parent information through the SCQ. The distribution between the diagnostic subgroups shows an overrepresentation of Pervasive Developmental Disorder-Not Otherwise Specified (PPD-NOS) in the participants under the age of 16 years, but there were no significant differences in the ASD symptoms as assessed by SCQ. We did not perform tests to estimate IQs for TD, but the parents of our control group reported no learning problems or psychiatric problems, and they were recruited from school children with normal school performance. Individuals with classical autism typically have significantly lower verbal IQs compared to performance IQs, although this varies within the ASD group. This situation also makes it challenging to match a control group [75]. We used the BRIEF, a parent-report measure, as a description of the presence of executive dysfunction in the participants. Research indicates that disagreement exists between performance-based tests and parent-report measures of EF [76]. We used parent-report BRIEF for all participants even though some of them were over 18 years old. This is because we had information that all participants still lived with their parents and we wanted to use the same BRIEF questionnaire across age groups. Performance-based measurements of EF could have contributed to a broader evaluation of executive dysfunction in the participants.

## Conclusion

Our findings of aberrant ERP signals in ASD during the passive part of a cued Go-NoGo task suggest altered visual perception (delayed N1) and increased neural activation related to attention allocation (enhanced P3a). Both components correlate significantly to the Behavioral Regulation Index of the BRIEF, suggesting relevance for real-life dysfunctions. The occipital N1 latency showed a fairly high sensitivity and specificity for the ASD diagnosis. Together, our results suggest that abnormal attention allocation, atypical control of alertness and “hyper-awareness,” are key pathophysiological features of ASD. Further, the results indicate that assessments during the passive parts of testing is needed to reveal important information of core neuropathology of ASD.

## Additional file

Additional file 1: **Table S1.** Demographics; number (*n*) and mean ± SD. **Table S2.** ERPs in TD compared with ASD without comorbid ADHD (ASD-ADHD). **Table S3.** Partial correlations of ERPs in participants without ADHD with BRIEF included subscales, adjusted for age. **Table S4.** ERPs in other conditions and recordings from other electrodes. **Figure S1.** Task stimuli. **Figure S2.** Relation between N1 latency and the Behavioral Regulation Index (BRI) including ASD subtypes. **Figure S3.** Relation between P3a amplitude and the Behavioral Regulation Index (BRI) including ASD subtypes. (DOCX 390 kb)

## Abbreviations

ADHD: Attention deficit disorder with or without hyperactivity
ADI-R: Autism Diagnostic Interview-Revised
ASD: Autism spectrum disorder
BRI: Behavioral Regulation Index
BRIEF: Behavior Rating Inventory of Executive Functions
EEG: Electroencephalogram
EF: Executive functions
ERP: Event-related potentials
FIQ: Full-scale intelligence quotient
GEC: Global Executive Composite
IQ: Intelligence Quotient
MI: Metacognition Index
PDD-NOS: Pervasive Developmental Disorder-Not Otherwise Specified
PIQ: Performance intelligence quotient
RT: Reaction time
RTV: Intra-individual reaction time variability
SCQ: Social Communication Questionnaire
SD: Standard Deviation
TD: Typical Developing
VIQ: Verbal Intelligence Quotient
