# Supplementary material for: Atypical event-related potentials revealed during the passive parts of a Go-NoGo task in autism spectrum disorder: a case-control study
Source: Mol Autism. 2019 Mar 5;10:10. doi: 10.1186/s13229-019-0259-3 (PMC6402134; doi:10.1186/s13229-019-0259-3)
Supplement: Supplementary file 1 — Table S1. Demographics; number (n) and mean ± SD. Table S2. ERPs in TD compared with ASD without comorbid ADHD (ASD-ADHD). Table S3. Partial correlations of ERPs in participants without ADHD with BRIEF included subscales, adjusted for age. Table S4. ERPs in other conditions and recordings from other electrodes. Figure S1. Task stimuli. Figure S2. Relation between N1 latency and the Behavioral Regulation Index (BRI) including ASD subtypes. Figure S3. Relation between P3a amplitude and the Behavioral Regulation Index (BRI) including ASD subtypes. (DOCX 390 kb) [file 13229_2019_259_MOESM1_ESM.docx]

**Additional file 1**

**Table S1. Demographics; number (n) and mean ± SD**

|  | **TD** | | **ASD** | |
| --- | --- | --- | --- | --- |
|  | n = 49 |  | n = 49 |  |
| **Gender** |  |  |  |  |
| Male | 31 |  | 36 |  |
| Female | 18 |  | 13 |  |
| **Age - years** |  |  |  |  |
| All | 49 | 15.6 ± 1.8 | 49 | 15.6 ± 2.4 |
| < 16 years | 27 | 14.3 ± 1.0 | 26 | 13.7 ± 1.3 |
| ≥ 16 years | 22 | 17.3 ± 1.1 | 23 | 17.8 ± 1.3 |
| **IQ** |  |  | 49 |  |
| Full scale IQ |  |  | 36 | 91.9 ± 17.7 |
| Verbal IQ |  |  | 47 | 87.6 ± 19.0 |
| Nonverbal IQ |  |  | 48 | 98.1 ± 19.3 |
| **SCQ** | 47 | 1.9 ± 2.3 | 49 | 18.7 ± 6.7 |
| Infantile Autism |  |  | 13 | 19.7 ± 6.0 |
| Asperger disorder |  |  | 18 | 17.7 ± 6.9 |
| PDD NOS |  |  | 18 | 19.0 ± 7.1 |
| **BRIEF** |  |  |  |  |
| Total score | 36 | 42.0 ± 6.0 | 37 | 67.6 ± 10.2 |
| BRI | 36 | 41.9 ± 3.6 | 38 | 68.0 ± 12.2 |
| MI | 36 | 43.0 ± 7.0 | 37 | 65.7 ± 9.9 |

Intelligence Quotient, **IQ**

**S**ocial **C**ommunication **Q**uestionnaire, **SCQ**

**B**rief **R**ating **I**nventory of **E**xecutive Function, **BRIEF**, Behavior Regulating Index, **BRI**, and Metacognition Index, **MI**

**Table S2. ERPs in TD compared with ASD without comorbid ADHD (ASD-ADHD)**

|  |  | **TD**  **n = 49** | **ASD – ADHD**  **n = 32** |  |  |
| --- | --- | --- | --- | --- | --- |
|  |  | **Mean (SD)** | **Mean (SD)** | ***p* - value** | **Cohens *d*** |
| After non-target S1  (S1 = plant) | **N1 amplitude** | 2.30 (4.86) | 1.89 (4.61) | 0.70 | 0.09 |
|  | **N1 latency** | 168.3 (15.7) | 182.6 (18.6) | < 0.001* | 0.83 |
|  | **Mean P3a Cz** | -0.99 (1.91) | 0.14 (1.81) | 0.009* | 0.61 |
| After non-target S2  (S1 *and* S2 = plant) | **N1 amplitude** | 2.37 (5.42) | 1.92 (4.44) | 0.714 | 0.09 |
|  | **N1 latency** | 165.4 (16.5) | 181.6 (17.5) | < 0.001* | 0.95 |
|  | **Mean P3a Cz** | -0.86 (1.87) | 0.26 (1.81) | 0.009* | 0.61 |

Latency in milliseconds, Amplitude in uV

* Significant at 0.01-level

**Table S3. Partial correlations of ERPs in participants without ADHD with BRIEF included subscales, adjusted for age**

|  | **BRIEF total** | **BRI** | **MI** |
| --- | --- | --- | --- |
| **n** | 59 | 58 | 58 |
| **N1 latency** | 0.30 | 0.38* | 0.25 |
| **P3a** | 0.26 | 0.37* | 0.18 |

* Significant at 0.01-level

**BRIEF** **total** score and subscales Behavior Regulation Index**, BRI**, and Metacognition Index, **MI**

**Table S4. ERPs in other conditions, and recordings from other electrodes.**

|  |  | **TD** | **ASD** |  |  |
| --- | --- | --- | --- | --- | --- |
|  |  | **Mean (SD)** | **Mean (SD)** | ***p* - value** | **Cohens *d*** |
| After non-cue S1 with pictures of emotional faces | **N1 amplitude** | -1.12 (4.74) | -0.27 (4.91) | 0.39 | 0.18 |
|  | **N1 latency** | 154.3 (19.9) | 169.2 (25.8) | 0.002* | 0.65 |
|  | **Mean P3a Cz** | -0.20 (1.90) | 0.93 (1.91) | 0.004* | 0.59 |
| After non-cue S1  (S1 = plant) | **Mean P3a Cz** | -0.99 (1.91) | 0.25 (1.99) | 0.002* | 0.64 |
|  | **Mean P3a Fz** | -2.57 (1.71) | -1.94 (1.92) | 0.085 | 0.35 |
|  | **Mean P3b Pz** | 2.43 (1.79) | 2.86 (2.37) | 0.31 | 0.20 |
| After *passive*  S2  (S1 *and* S2 = plant) | **Mean P3a Cz** | -0.86 (1.87) | 0.35 (2.46) | 0.007* | 0.55 |
|  | **Mean P3a Fz** | -3.00 (2.11) | -1.54 (1.96) | 0.001* | 0.72 |
|  | **Mean P3b Pz** | 2.28 (2.08.) | 1.74 (2.16) | 0.21 | 0.25 |

* Significant at 0.01-level

Latency in milliseconds, Amplitude in uV

Cz: Central Midline electrode

Fz: Frontal Midline electrode

N1 is recorded from occipital electrodes O1 and O2 and averaged between these

The test, ECPT, with emotional faces from Ekman [1] is described in detail in a previous paper by Høyland et al. [2]

**FigS1. Task stimuli**

| **Time in milliseconds** | | 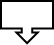  100  1500  1000  300  100 | |
| --- | --- | --- | --- |
| **S1** | **S2** | 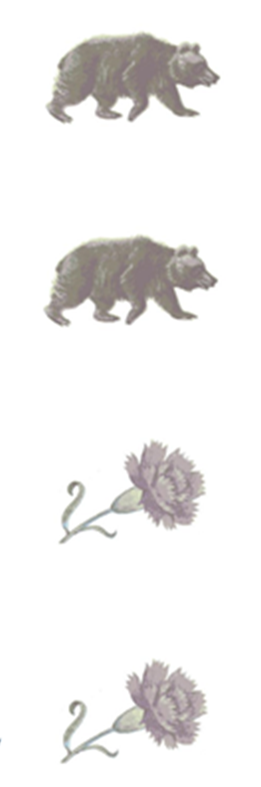 | 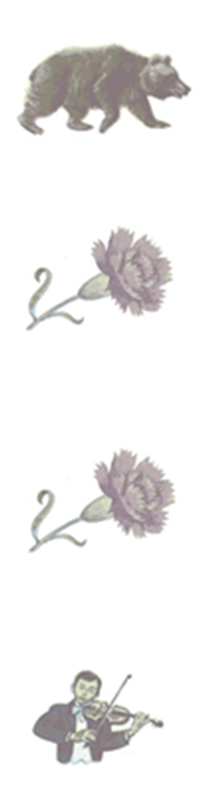 |
| **Target-condition** | **Go** |  |  |
|  | **NoGo** |  |  |
| ***Non-*target-condition** | ***Non-*target** |  |  |
|  | With novelty sound - **excluded** |  |  |

**FigS2. Relation between N1 latency and the Behavior Regulating Index (BRI) including ASD subtypes**


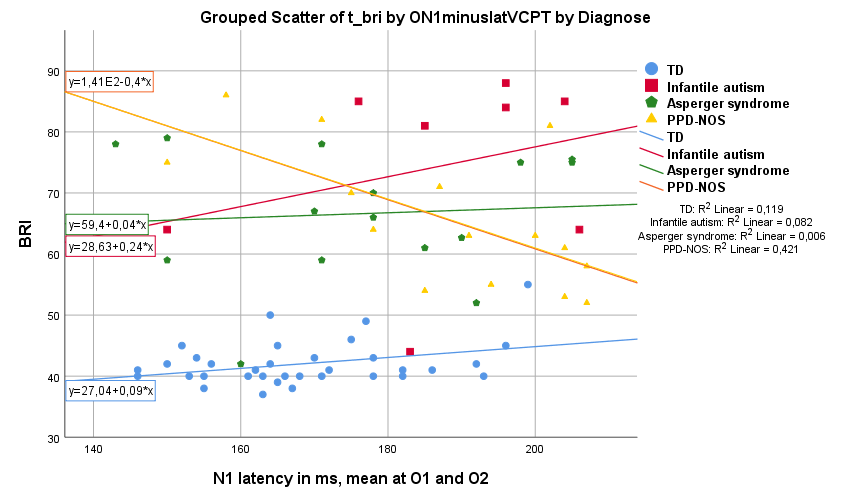


The relation between N1 latency (mean of electrodes O1 and O2) in milliseconds (ms) and the Behavior Regulating Index (BRI), an index of the Behavior Rating Inventory of Executive Function (BRIEF). The subtypes of ASD are marked with different markers/ colors to illustrate how the relation between the ERP component and BRI measure differed. Individuals with PDD-NOS show a different ERP/BRI relation than other ASD subtypes.

**FigS3.** **Relation between P3a amplitude and the Behavior Regulating Index (BRI) including ASD subtypes**

**
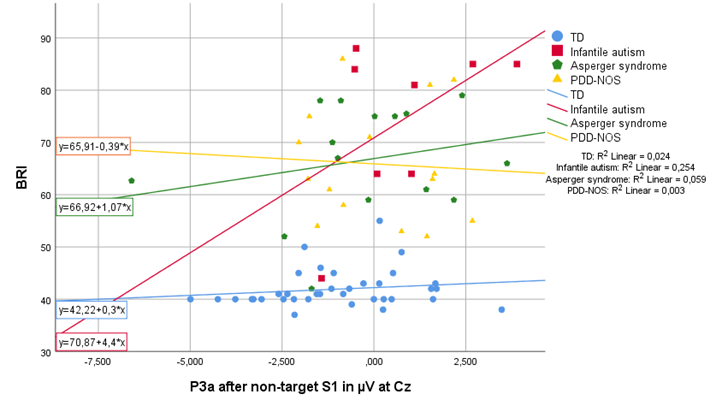
**

The relation between P3a amplitude at the electrode Cz and the Behavior Regulating Index (BRI), an index of the Behavior Rating Inventory of Executive Function (BRIEF). Subtypes of ASD are marked with different markers/ colors to illustrate how the relation between the ERP component and BRI measure differed. Individuals with PDD-NOS show a different ERP/BRI relation than other ASD subtypes.

**References**

1. Ekman P, Friesen WV: **Pictures of facial affect**: consulting psychologists press; 1975.

2. Hoyland AL, Naerland T, Engstrom M, Lydersen S, Andreassen OA: **The relation between face-emotion recognition and social function in adolescents with autism spectrum disorders: A case control study**. *PLoS One* 2017, **12**(10):e0186124.
